# Antipyretics

**Published:** 1894-06

**Authors:** W. A. Archer

**Affiliations:** Houston, Texas


For Texas Medical Journal.

### ANTIPYRETICS.

BY DR. W. A. ARCHER, OF HOUSTON, TEXAS.

Read before Houston Medical Society.

MR. CHAIRMAN AND GENTLEMEN:—Appointed your essayist for this evening, I present, for your consideration, the subject—Antipyretics.

Antipyretics are agents by which we are enabled to reduce the temperature of a patient who is in a state of pyrexia; i. e., whose temperature is above the normal. They may be divided into two great classes: Those that are taken internally and by acting through the system produce a defervescence, and those that cause the dissipation of heat by applications to the surface.

I propose to-night to confine my remarks to the former class with one exception, to be hereafter noted.

Only a few years ago, comparatively, it was announced that a substance had been discovered, or prepared, which, if given to a person with fever, would cause a rapid decline of the fever.

The oldest members of the profession, present, remember the incredulity at first, and afterwards the astonishment which took possession of the professional mind when Knorr's Antipyrin was first introduced and its properties demonstrated—coming in the guise of quackery, a proprietory preparation—it was eyed askance, but forced notice of its presence by its undoubted effects, and was soon accepted by all classes of the profession, as the "supplying of the long-felt want" of how to control and modify fever. The great body of the profession was delighted; considered it one of the greatest advances that had ever been made in medicine.

Quickly, investigators presented other substances with similar properties, many of which were found too dangerous or uncontrollable to use. Some were found to be failures and some frauds, until the chief ones that have survived the crucible tests of experience, are the original Antipyrin, Phenacetine, and Acetanilide, called at first Antifebrin. These are about all that are extensively used at this time.

There is a new candidate for favor just now, called Guaiacol, which acts by making local applications to circumscribed portions of the surface of the body; said to be non-poisonous and very effective. Thirty drops rubbed upon the surface of the body are said to be sufficient to reduce the temperature in fever, to, or near, the normal. Too large doses produce subnormal temperature, and are not to be used. These statements, endorsed by Da Costa, *must* have attention from the profession. This is the exception mentioned above in the division of Antipyretics into external and internal.

Now let us see how they have fulfilled the promises held out at the time of their introduction.

Two theories have been advanced to account for high temperatures in the body. They are, that the heat processes of the body are over-estimulated, and that an excess of heat is generated and so heats the patient above the normal. The other, that no more heat is generated in the body, but that the processes by which the heat is dissipated, are interrupted and so the heat accumulates. Each has its advocates and they base therapeutics upon their theories. Neither has been able to prove the theory advocated, and the truth is probably a mixture of the two theories, with most of the causation of fever depending upon the theory of excessive heat production. How the internal Antipyretics act to dissipate this heat has not been determined, but it has been found that their action is evanescent, and in the large majority of cases ultimately injurious. It is very alluring to the physician, the knowing that with a few doses of an Antipyretic he can "cool dowu" a fever and make the patient and his friends glad that it is gone.

But, alas, the fever will surely return, and instead of being better, your patient is worse than before. The disease is more thoroughly fastened upon him,—his power of resistance is lessened,—the crasis of the blood is lowered. The time which should have been spent in correcting the perverted action of .the depurating and eliminative apparatus has been lost, while the depraving processes of the disease have continued to act, not only unchecked, but actually assisted in their attack upon the constitution of the patient, by the lowering effects of the antipyretic. Therefore I say that in fevers *generally* it is best to avoid the extensive use of the internal antipyretics. However, sometimes there are fevers of an extremely intense grade, where the effects of the hyperpyrexia are destructive and threatening *per se*, and do not give time for remedies to produce their effects. Then, the antipyretic comes as a veritable godsend—it reduces the temperature, which is the subject most urgent, and thus affords time to meet other indications. Here is probably the most important application of antipyretics, *as* antipyretics—I say *as* antipyretics, because of the fact that these remedies have another and most striking set of properties, besides their effects as antipyretics, strictly. They act as analgesics, and *these* properties have caused them to be abused even more than their antipyretic properties have. The profession was as much astonished by the discovery of their analgesic effects as it had been by their first discovery. These powers were heralded abroad by the press, both sectarian and non-sectarian, till the laity became acquainted with them to a very large extent, and the consumption of them without professional advice has become something enormous. Quackery came foward and appropriated them, and now rings the changes upon various combinations of them with the various algias, headache cures, and the like, Many physicians have been beguiled into lending their aid in popularizing the use of these proprietary preparations, so that now every newly graduated pharmacy student is ambitious of getting up and disseminating some *so-called harmless* panacea and pain reliever with a high-sounding name. Druggists, manufacturing, wholesale and retail, are forming their stock-companies to make these nostrums, and are overwhelming the physicians with their importunities to prescribe

*their* preparations, each claiming that his preparation is not a mere mixture but is a scientifically made chemical combination. They are generally made by mixing together caffeine, soda and acetanilide (on account of its cheapness).

They are all injurious in many cases, and their indiscriminate use should be prevented as far as possible.

Antipyretics are exceedingly valuable as analgesics, but their use should be restricted to suitable cases selected by the physician, and the limits to their use carefully indicated. Sometimes when they are indicated, a change from one to another is followed by good results. Too much use of these remedies will produce anæmias and cachexsias, which are dangerous to pregnant women and probably injurious to the fœtus. Besides, they probably predispose to post partem hemorrhage and exercise other direct and remote deleterious effects upon the pregnant and parturient woman; so, should be prescribed for such patients with much reserve.

To sum up, then, Antipyretics are an exceedingly valuable acquisition to therapeutics, but should be used only upon clear indications, with care to suspend their use promptly as soon as their objects are accomplished, or when they begin to produce deleterious results, whether the objects aimed at are accomplished or not. When used with these limitations they will not be such a *doubtful* blessing in therapeutics.

